# Malignant hypertension induces thrombotic microangiopathy and renal failure: A case report

**DOI:** 10.1097/MD.0000000000041186

**Published:** 2025-01-10

**Authors:** Xi Sun, Chunying Liu, Yanyun Ren, Liqun He, Youhua Xu

**Affiliations:** a Faculty of Chinese Medicine, Macau University of Science and Technology, Taipa, Macau, China; b Affiliated Hospital of Shaanxi University of Traditional Chinese Medicine, Shaanxi, China; c Shuguang Hospital Affiliated to Shanghai University of Traditional Chinese Medicine, Shanghai, China.

**Keywords:** malignant hypertension, renal failure, thrombotic microangiopathy

## Abstract

**Rationale:**

Thrombotic microangiopathies (TMA) caused by malignant hypertension is an acute and critical disease among rare diseases. Although renal biopsy pathology is a golden indicator for diagnosing kidney disease, it cannot distinguish between primary and secondary TMA and requires a comprehensive diagnosis in conjunction with other laboratory tests and medical history.

**Patient concerns:**

A 33-year-old young man was hospitalized due to unexplained kidney failure.

**Diagnosis:**

A young man was admitted to the hospital with unexplained renal failure with severe heart failure and was diagnosed with TMA by renal biopsy. Further investigation excludes primary TMA and is considered to be due to malignant hypertension.

**Interventions:**

Temporary hemodialysis combined with aggressive blood pressure control and the use of angiotensin receptor-neprilysin inhibitor are the primary management approach.

**Outcomes:**

Upon discharge, the patient had ceased dialysis and showed significant renal function recovery. After 1 year follow-up period, creatinine levels remained stable at 2.373 mg/dL, while lactate dehydrogenase, B-type natriuretic peptide, and platelet levels all returned to within normal ranges.

**Lessons:**

TMA caused by malignant hypertension is rare and similar to the clinical manifestations of TMA caused by Thrombotic Thrombocytopenic Purpura and Hemolytic Uremic Syndrome, but it is often sudden and critical. Early identification and diagnosis and aggressive antihypertensive therapy, must be given to avoid end-stage renal disease, long-term maintenance of dialysis, and the financial burden of unnecessary plasmapheresis and potential side effects of glucocorticoids.

## 1. Introduction

Thrombotic microangiopathies (TMA) are a cluster of clinical syndromes characterized by microangiopathic hemolytic anemia, thrombocytopenia, and organ damage resulting from microcirculatory thrombosis. The most prototypical diseases include thrombotic thrombocytopenic purpura (TTP), hemolytic uremic syndrome (HUS), and atypical HUS (aHus).^[1]^ Moreover, TMA can also occur in malignant hypertension, drugs (such as quinine, cyclosporine, tacrolimus, etc), tumors, transplantation, viral infections, pregnancy-related, postpartum HUS, HIV infection, rheumatic immune diseases, such as systemic lupus erythematosus, etc.^[2]^ TMA caused by malignant hypertension exhibits similar clinical manifestations to classic TMA and should be meticulously differentiated. Their treatment methods and prognosis are entirely distinct. Early and accurate diagnosis can prevent unnecessary plasma exchange, and proactively controlling blood pressure to reach the target will aid patients in recovering their renal function. This article presents a case of malignant hypertension complicated by TMA, leading to renal failure. After treatment, the patient’s renal function partially recovers, and the patient was weaned from dialysis, aiming to enhance clinicians’ differential diagnosis of TMA.

## 2. Case presentation

The patient, a 33-year-old young man, was admitted to the hospital on January 8, 2023 with “increased nocturia for 2 months and abnormal renal function for 1 day.” Patients have no apparent cause of nocturia before 2 months, 3 to 4 times per night. On January 6, 2023, due to nausea and vomiting, blood pressure of 210/150 mm Hg (1 mm Hg = 0.133 kPa) was measured in the emergency department of the first hospital. Laboratory tests revealed the following results: hemoglobin (Hb) level of 95 g/L (reference range: 130–175 g/L), platelet (PLT) count of 96 × 10^9^/L (reference range: 125–350 × 109/L), serum creatinine (Scr) level of 6.49 mg/dL (reference range: 0.644–1.096 mg/dL), blood urea nitrogen (BUN) 65.824 mg/dL (reference range: 8.683–22.408 mg/dL), uric acid (UA) 12.466 mg/dL (reference range: 3.494–7.19 mg/dL). The patient was advised to be hospitalized, but the patient refused, so he went to the emergency department of the second hospital, and the laboratory tests showed that the Scr level was 7.797 mg/dL, BUN level was 65.824 mg/dL, the UA was 12.146 mg/dL, the estimated glomerular filtration rate was 8.21 mL/min/1.73 m^2^ (reference range: >80 mL/min/1.73 m^2^) and lactate dehydrogenase (LDH) 824 U/L (reference range: 120–250 U/L). The patient was eventually admitted to our department through the emergency department (January 8, 2023). At the time of admission, the patient was clinically manifested as chest tightness and shortness of breath, inability to lie flat at night, nausea, and nocturia. He has a history of hypertension for 2 years, has not used any antihypertensive drugs, and denies a previous history of chronic diseases such as diabetes and coronary heart disease. Physical examination on admission: blood pressure 210/130 mm Hg. Wet rales can be heard in both lungs on auscultation, the heart boundary is enlarged to the left, the heart rate is 102 beats per minute, and there is no edema in both lower extremities.

Laboratory tests revealed the following results: Scr levels of 7.82 mg/dL, BUN level of 75.627 mg/dL, UA level of 12.331 mg/dL, PLT of 115 × 109/L, Hb level of 90 g/L, while the albumin (ALB) level of 38.0 g/L(reference range: 40–55g/L), LDH level of 902 U/L, B-type brain natriuretic peptide level of 1018 pg/mL (reference range: 0–100 pg/mL), 24-hour urine protein quantification level of 1610 mg/24 h (reference range: 28–141 mg/24 h), urinary ALB to creatinine ratio level of 1127 mg/g (reference range: <30 mg/g), urinary β2 microglobulin level of 3.10 mg/L(reference range: 0.1–0.3 mg/g), parathyroid hormone level of 23.47 pmol/L (reference range:1.58–6.03 pmol/L), ferritin level of 747.80 ng/mL (reference range: 4.63–204 ng/mL), renin level of 295.64 pg/mL (reference range: 4–24 pg/mL), aldosterone level of 240.82 pg/mL (reference range: 10–160 pg/mL). The patient’s myocardial injury markers, autoantibodies, hemorrhagic fever antibodies, anti-neutrophil cytoplasmic antibodies, anti-phospholipase A2 receptor, anti-glomerular basement membrane antibody, serum immunofixation electrophoresis, serum free light chain and ratio, thyroid function, coagulation, and routine stool tests were all within normal ranges.

Cardiac color ultrasound examination revealed left ventricular hypertrophy, with increased interventricular septum and left ventricular wall thickness. Moderate pulmonary artery hypertension was also observed. The ejection fraction of the heart was measured at 53%. The ultrasound of the kidneys revealed that the right kidney measured 105 × 56 × 44 mm, with a cortex thickness of approximately 9 mm. The left kidney was found to be 120 × 55 × 48 mm in size, with a cortex thickness of around 8 mm. Both kidneys exhibited roughly regular shapes, non-smooth surfaces, and evidence of diffuse damage. A chest computed tomography scan revealed a small pericardial effusion and a minimal pleural effusion in the left pleural cavity. Ophthalmology consultation results showed hypertension retinopathy in both eyes.

Levamlodipine Besylate Tablets (5 mg, po, bid), nifedipine controlled-release tablets (0.03 g, po, bid) and carvedilol (10 mg, po, bid) were given for antihypertensive therapy since January 8, 2023. Erythropoietin (3000 IU, sc, tiw) was administered to correct anemia, and sodium bicarbonate tablets (1.5 g, po, bid) were used to correct acidosis.

The cause of kidney failure in this patient is unknown. Renal biopsy was not performed because the patient was currently severely hypertensive with severe heart failure and was unable to lie flat. The patient’s Scr continued to rise to 9.605 mg/mL, and urea nitrogen increased to 84.03 mg/mL, which met the dialysis standard (January 16, 2023). Although there were no clear signs of considerable renal atrophy, the patient’s kidney function persistently deteriorated, accompanied by symptoms of nocturia and a modest amount of protein in the urine. After obtaining the patient’s consent, sacubitril/valsartan (100 mg, po, bid) was given, and dialysis treatment was conducted simultaneously (January 18, 2023). What puzzled us was that the urine output of patients increased significantly, from <1000 mL/day at the time of admission to 2000 mL/day, but the improvement of renal function was not obvious. After the patient’s blood pressure was maintained at about 130/85 mm Hg and hemodialysis was performed for 1 week, a renal biopsy was performed, and dialysis was suspended after the biopsy (January 29, 2023).

**Figure 1.**
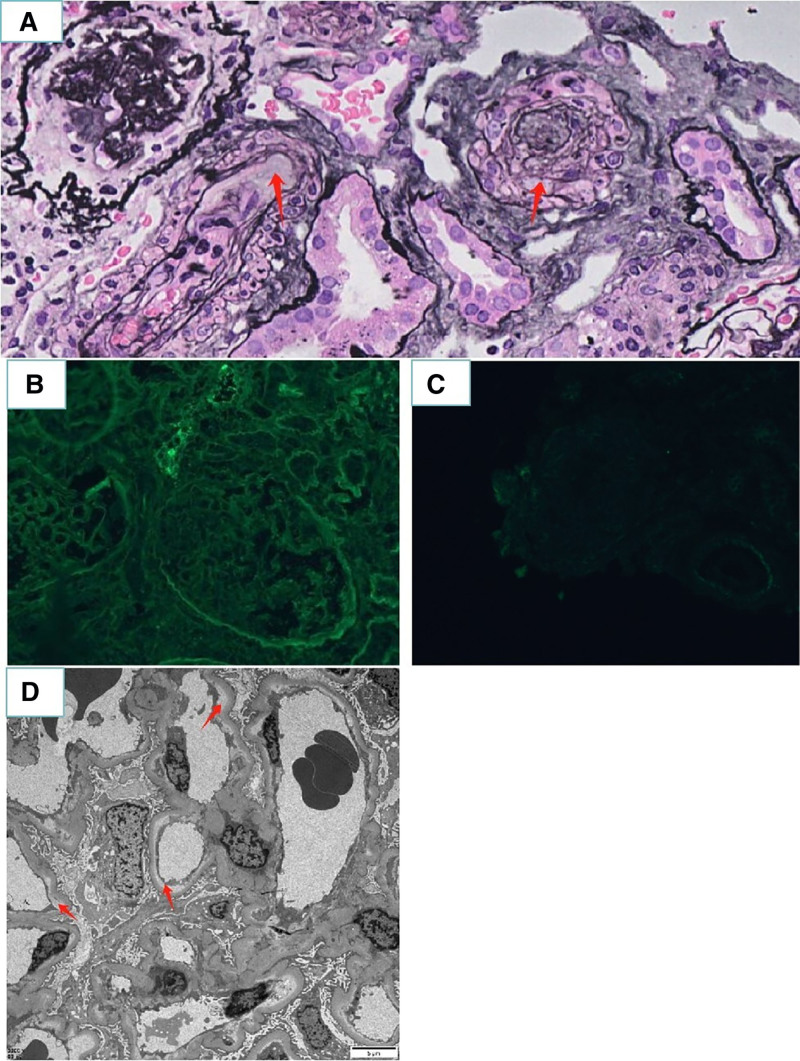
Kidney biopsy. (A) Periodic-acid silver methenamine staining shows arteriole wall thickening, mucinous degeneration, and intimal onion-like hyperplasia, leading to official lumen stenosis, occlusion (×200). (B and C) Immunofluorescent images of frozen sections are negative for immunoglobulin and complement. (D) Electron microscopy shows segmental thickening of the glomerular basement membrane, widening of some subendothelial spaces with double-track signs. Scale bar = 5 μm.

The pathological analysis of renal biopsy showed that the patient was consistent with thrombotic microangiopathy, primarily involving renal arterioles, and should be noted to exclude malignant hypertensive renal injury in combination with clinical examination (January 30, 2023). The renal puncture tissue was routinely stained with hematoxylin and eosin, Periodic Acid-Schiff staining, Periodic Acid-Silver Methenamine and Masson. The findings included 12 glomeruli, 3 of which showed ischemic sclerosis. The fluorescent slides were stained with Periodic Acid-Silver Methenamine, revealing 9 glomeruli, 5 of which had glomerulosclerosis. The remaining glomerular mesangial cells and matrix showed slight proliferation, with segmental thickening of the basement membrane and the formation of a double-track sign. No spike-like structure was observed, and periglomerular fibrosis was seen in some glomeruli. Mild renal interstitial edema, along with plasma cell infiltration and fibrosis, arteriole wall thickening, mucinous degeneration, and intimal onion-like hyperplasia, leading to official lumen stenosis, occlusion, and occasional thrombosis. Immunofluorescence showed that immunoglobulin G, immunoglobulin M, immunoglobulin A, complement Component 3, complement component 1 q were all negative, and immunofluorescence revealed no immune complex deposition. Electron microscopy revealed segmental thickening of the glomerular basement membrane, widening of some subendothelial spaces with double-track signs, and fusion of most of the foot processes. No definite electron dense material deposition was found. The pathological diagnosis results of renal puncture were consistent with thrombotic microvascular lesions, mainly renal arteriole lesions. Please combine it with clinical practice and be careful to exclude malignant hypertensive renal damage (Fig. 1). Further testing of von Willebrand factor-cleaved protease (a disintegrin and metalloproteinase with thrombospondin motif, member 13, ADAMTS13) showed a result of 121.25% (reference range: 42.16–126.37%), and peripheral blood were normal (February 2, 2023).

Combined with the patient’s history of hypertension and pathological analysis of renal biopsy, as well as other laboratory tests, we believe that the patient’s TMA is secondary to malignant hypertension rather than primary TMA (February 3, 2023).

Before discharge, the Scr level decreased to 4.972 mg/dL, the BUN level decreased to 56.02 mg/dL, the Hb level increased to 125 g/L, and the PLT count increased to 281 g/L, both of which returned to normal levels. The 24-hour urine protein quantification decreased to 924 mg/24 h, and the B-type brain natriuretic peptide decreased to 132 pg/mL, LDH drops to 300 U/L. Post-discharge regimen: sacubitril/valsartan (100mg, po, bid), nifedipine controlled-release tablets (0.03 g, po, bid) and carvedilol (10 mg, po, bid) were given for antihypertensive therapy.

Follow-up results: 2 weeks after discharge, the Scr level was 4.87 mg/dL, the BUN level was 61.622 mg/dL, ALB increased to 45.0 g/L, the LDH dropped to 283 U/L, and the PLT count was 232 × 10^9^/L. Follow-up results 2 months after discharge: Scr dropped to 3.56 mg/dL, BUN dropped to 36.693 mg/dL, and LDH was normal. Six-month follow-up results: Scr dropped to 2.712 mg/dL, and BUN dropped to 31.791 mg/dL (August 2, 2023). After 1 year of follow-up, Scr dropped to 2.373 mg/dL and BUN dropped to 25.2 mg/dL (February 10, 2024).

## 3. Discussion

This patient was diagnosed with TMA by renal biopsy, and the underlying cause of the TMA remains to be further elucidated. The patient in question is a young male with a 2-year history of hypertension. He has not taken any antihypertensive medications in the past, and his blood pressure levels were not closely monitored. Upon admission to the hospital, his blood pressure significantly increased. Concurrently, the ophthalmology consultation revealed vision loss and fundus lesions, accompanied by acute kidney injury and kidney disease. The biopsy findings indicated thickening of the arterial wall, mucus degeneration, onion-like intimal hyperplasia, lumen stenosis, occlusion, and occasional thrombosis. Immunofluorescence analysis demonstrated no immune complex deposition. The pathological diagnosis was consistent with thrombotic microvascular disease, primarily involving renal arteriolar disease. In clinical practice, it is essential to carefully exclude the possibility of malignant hypertensive renal damage. Moreover, the patient’s lactate dehydrogenase level significantly increased upon admission, while the mild decrease in PLT was consistent with the diagnosis of TMA.

Based on the patient’s medical history and other laboratory test results, no abnormal findings were observed in the patient’s lying and standing aldosterone examination, adrenal computed tomography scan, renal ultrasound, and vascular examination, which did not support primary aldosteronism, renal artery stenosis, or pheochromocytoma. Additionally, secondary hypertension caused by hypercortisolism can be ruled out. The patient was diagnosed with acute renal failure. In conjunction with autoantibodies, anti-neutrophil cytoplasmic antibodies-associated vasculitis antibodies, and normal anti-glomerular basement membrane antibodies, rheumatic immune diseases such as systemic scleroderma, systemic lupus erythematosus, and primary antiphospholipid antibody syndrome can be excluded. Acute renal failure and TMA secondary to symptoms, systemic vasculitis, rapidly progressive glomerulonephritis, and TMA caused by drugs, tumors, etc. can also be ruled out.

Before being admitted, the patient’s diastolic blood pressure was >130 mm Hg, platelet count was greater than 50 × 10^9^/L, and the ADAMTS13 enzyme activity test was negative. In addition to hypertension, this patient had no other clear precipitants for TMA. Furthermore, the 24-hour urine protein quantification on admission was 3.86 g/24 h. After effective control of blood pressure and no plasma exchange, the 24-hour urinary protein quantification decreased to 0.09 g/24 h. At the same time, the platelet count returned to normal, and lactate dehydrogenation occurred in about 4 weeks. Enzyme levels returned to normal, and the patient was disconnected from dialysis. In this patient’s renal pathology, only ischemic changes were observed in the glomeruli, and immunofluorescence was completely negative. It was considered that the proteinuria was caused by hypertension, rather than renal hypertension caused by underlying renal disease. These evidences support that the TMA changes in this patient were caused by malignant hypertension. The basis of malignant hypertension is essential hypertension. Although many examination indicators have improved significantly, the patient’s Scr still remains at approximately 2.71 mg/dL. Considering that the patient’s blood pressure was very high at the onset of the disease and the duration is long, the vascular lesions are not easy to fully recover, and the results of further follow-up are still awaited.

While TMA induced by malignant hypertension presents similar clinical manifestations to TMA caused by classic TTP, HUS, and aHus, its management and prognosis differ significantly. Therefore, it is essential to carefully distinguish between these conditions to avoid inappropriate glucocorticoid and plasma exchange therapy.

TTP is a rare and life-threatening diffuse thrombotic microangiopathy, characterized by microangiopathic hemolytic anemia, severe thrombocytopenia, and organ damage (such as kidneys, central nervous system, etc) caused by microthrombosis, which is its main pathological feature.^[3]^ TTP is associated with severe defects in ADAMTS13.^[4]^ The first acute episode of TTP usually occurs in adulthood, and the main cause is anti-ADAMTS13 autoimmune disease. In the past, fever, microangiopathic hemolytic anemia, thrombocytopenia, neurological symptoms, and renal insufficiency were known as the clinical “five syndromes” of TTP.^[5]^ In terms of treatment, plasma exchange is the basic treatment for TTP. Once TTP is highly suspected, plasma exchange should be performed as soon as possible.^[3]^ Glucocorticoids can stabilize platelet and endothelial cell membranes and inhibit the production of ADAMTS13 antibodies. It is generally recommended to combine treatment with plasma exchange at the same time.

HUS is further classified by its cause: infection-related HUS, including *Streptococcus pneumoniae*, *Escherichia coli*, influenza virus, HIV, and H1N1 virus infection, etc, nontypical HUS, including diacylglycerol kinase ε mutation-related sex, complement dependence, vitamin C deficiency, etc. Secondary HUS can be secondary to autoimmune diseases, drugs, tumors, malignant hypertension, pregnancy, bone marrow transplantation, organ transplantation, and kidney disease.^[6]^ The typical clinical manifestations of HUS are the triad: microvascular hemolysis, thrombocytopenia, and acute kidney injury,^[7]^ with kidney involvement generally being the most severe. Treatment includes antimicrobial therapy, fluid rehydration, renal replacement therapy, blood transfusion, and plasma exchange therapy,^[7]^ kidney transplantation/combined liver and kidney transplantation,^[8]^ anticomplement C5 monoclonal immunoglobulin, and eculizumab.^[9]^

TMA is one of the serious complications of malignant hypertension. The overall presentation may be similar to primary TMA. This case was confirmed by renal biopsy. Combined with medical history and other laboratory tests, primary TMA was excluded. The pathophysiological mechanism of TMA caused by malignant hypertension remains unclear. It may be related to the activation of the renin-angiotensin-aldosterone system and endothelial dysfunction,^[10]^leading to the formation of small blood vessels and intravascular thrombosis, red blood cell fragmentation, platelet hemolytic consumption, and elevated LDH.^[11,12]^ Persistent TMA is associated with endothelial injury, fibrinoid necrosis, and occlusive vasculopathy, which may explain persistent renal failure and dependence on dialysis.^[13]^

Although some research has demonstrated that malignant hypertension might be associated with a decline in multimer-cleaving proteases, the decrease is typically minimal, and plasma exchange is not recommended.^[13]^ Moreover, the platelet count typically remains at ≥50 × 10^9^/L. Conversely, the platelet count in typical HUS/TTP cases may drop as low as 20 × 10^9^/L.^[14]^ Paying particular attention to platelet counts may aid in the identification of TMA and help to avoid unnecessary plasma exchange therapy. In this particular case, the patient’s renal biopsy revealed TMA, and his medical history and laboratory test results did not corroborate HUS/TTP. Consequently, the patient received only antihypertensive and dialysis treatments. Consequently, the platelet count returned to normal, renal function partially recovered, and the patient was weaned from dialysis. Malignant hypertension frequently leads to kidney damage. If blood pressure is not effectively managed, the condition can rapidly worsen and potentially progress to uremia, necessitating dialysis or even resulting in death.

Sacubitril/valsartan is the first angiotensin receptor-neprilysin inhibitor that acts simultaneously on the natriuretic peptide system and the rein-angiotensin-aldosterone-system. While blocking the rein-angiotensin-aldosterone-system to prevent an increase in blood pressure, it enhances the natriuretic peptide system, exerting effects that reverse cardiac remodeling and improve heart failure. Besides its comprehensive antihypertensive effects, sacubitril/valsartan also provides excellent protection to target organs such as the heart, kidneys, and blood vessels. Sacubitril/valsartan can improve the estimated glomerular filtration rate and slow the progression of kidney disease; it also has anti-endothelial proliferation and antifibrotic effects, improves endothelial function, and delays and reverses plaque formation. Therefore, in patients with hypertension and cardio-renal comorbidities, sacubitril/valsartan can bring additional benefits.^[15]^

This study has certain limitations. Due to the small number of cases and large individual differences, which may lead to bias and limited reproducibility of results, we look forward to future large-sample, randomized controlled studies and experiments.

In conclusion, malignant hypertension can lead to TMA, which differs from classic HUS/TTP in terms of etiology. Although renal biopsy pathology is a golden indicator for diagnosing kidney disease, it cannot distinguish between primary and secondary TMA and requires a comprehensive diagnosis in conjunction with other laboratory tests and medical history. Early detection and identification are crucial to avoid unnecessary glucocorticoid and plasma exchange use. The application of sacubitril/valsartan is beneficial, and controlling blood pressure is key. Renal function can be partially restored, and some patients can be weaned off dialysis.

## Author contributions

**Data curation:** Xi Sun, Yanyun Ren.

**Funding acquisition:** Chunying Liu, Youhua Xu.

**Supervision:** Chunying Liu, Liqun He.

**Visualization:** Xi Sun.

**Writing – original draft:** Xi Sun.

**Writing – review & editing:** Liqun He, Youhua Xu.
